# The *Rb1* tumour suppressor gene modifies telomeric chromatin architecture by regulating TERRA expression

**DOI:** 10.1038/srep42056

**Published:** 2017-02-07

**Authors:** I. Gonzalez-Vasconcellos, R. Schneider, N. Anastasov, S. Alonso-Rodriguez, B. Sanli-Bonazzi, J. L. Fernández, M. J. Atkinson

**Affiliations:** 1 INIBIC-Complejo Hospitalario Universitario A Coruña, Unidad de Genética, As Xubias, 84, 15006-A Coruña, Spain; 2 Institut für Experimental Genetics, Helmholtz Zentrum München, Deutsches Forschungszentrum für Gesundheit und Umwelt Ingolstädter Landstr. 1, 85764 Neuherberg, Germany; 3 Institute of Radiation Biology, Helmholtz Zentrum München, Deutsches Forschungszentrum für Gesundheit und Umwelt Ingolstädter Landstr. 1, 85764 Neuherberg, Germany; 4 Laboratorio de Genética Molecular y Radiobiología, Centro Oncológico de Galicia, 15009-A Coruña, Spain

## Abstract

The tumour suppressor gene (*Rb1*) is necessary for the maintenance of telomere integrity in osteoblastic cells. We now show that the compaction of telomeric chromatin and the appropriate histone modifications of telomeric DNA are both dependent upon Rb1-mediated transcription of the telomere-derived long non-coding RNA TERRA. Expression of TERRA was reduced in Rb1 haploinsufficient cells, and further decreased by shRNA-mediated reduction of residual Rb1 expression. Restoration of Rb1 levels through lentiviral transduction was sufficient to reestablish both transcription of TERRA and condensation of telomeric chromatin. The human chromosome 15q TERRA promoter contains predicted retinoblastoma control elements, and was able to confer Rb1-dependent transcription upon a promoterless reporter gene. Chromatin immunoprecipitation revealed preferential binding of phosphorylated over non-phosphorylated Rb1 at the TERRA promoter. As *Rb1*-deficient cells show increased genomic instability we suggest that this novel non-canonical action of Rb1 may contribute to the tumour suppressive actions of *Rb1*.

Osteosarcoma are unusual amongst solid tumours in having a high rate of genomic instability and a propensity for developing at sites of prior radiation exposure^1^,^2^. Mutation of the *Rb1* tumour suppressor gene contributes to both of these phenotypic features. Thus, germ-line mutation of *RB1* increase the risk of radiation-induced osteosarcoma^3^,^4^, while inheritance of a haploinsufficient *Rb1* allele or deletion of an *Rb1* allele in the osteoblastic lineage is sufficient to predispose mice to radiation-induced osteosarcoma^5^. *Rb1* dependent radiation-induced osteosarcoma show increased chromosome instability that is coupled with accelerated telomere shortening in dividing cells^4^.

A number of proteins co-localize with telomeric DNA to create the shelterin complex. The correct assembly of shelterin is essential for the organization and stability of telomeric heterochromatic chromatin and the maintainance of telomere length^6^. These actions are essential to prevent chromosomal ends from being recognised as DNA double strand breaks and a resultant triggering of an inappropriate DNA damage response. In its heterochromatic form telomeric chromatin contains histone octamers that are enriched for the hypermethylated lysine residues H3K9me3 and H4K20me3^6^,^7^.

The long non-coding RNA TERRA (Telomere-Repeat-Containing-RNA) is an RNA polymerase II transcript from a genomic locus that extends distally from the subtelomeric region into the telomere^8^,^9^,^10^. TERRA transcripts contain long stretches of transcribed telomeric repeats although the extent of the full length transcripts remains unclear^10^. In common with other long-non-coding RNAs^11^,^12^ the TERRA RNA is able to co-localize with its transcription site causing it to accumulate at the telomeres^10^,^13^, where TERRA forms hybrid DNA:RNA structures (R-loops)^14^,^15^. The TERRA R-Loops may be instrumental in building the shelterin complex as TERRA can form RNA:Protein complexes with both TRF1 and TRF2^14^. Members of the hnRNP family are also bound by TERRA, suggesting that these may anchor TERRA to specific chromatin sites^16^. Intriguingly, TERRA has also been reported to physically interact with the histone methyltransferase SUV39H1 that promotes the telomere histone H3K9 methylation within telomere nucleosomes^17^.

The Rb1-dependent telomeric stability, the interaction between RB1 and SUV39H1^17^, as well as our recent demonstration that the lncRNA PARTICLE forms promoter DNA:RNA hybrids that result in local regulates local epigenetic marks^18^, suggest that TERRA may be acting as an Rb1-dependent modulator of telomeric epigenetics. We now show that *Rb1* indeed influences telomeric integrity through directly regulation of TERRA promoter Rb1-dependent modulation of TERRA transcript levels and that ultimately results in structuring of the telomere architecture.

## Materials and Methods

### Primary osteoblast culture

Osteoblasts were grown from explants of long bone fragments from 2- to 4-week-old FVB-COL1A1-Cre-Tg × FVB Rb1^+/loxP^ or FVB-COL1A1-Cre-Tg × FVB Rb1^+/+^ mice as described previously^19^. All animals were housed and sacrificed using facilities approved by the animal welfare committee of the State of Bavaria. Breeding, sacrifice and organ removal from mice was conduced with the approved of the responsible state authority (Regierung von Oberbayern license number 55.2-1-54-2532-113-2). All experiments were performed in accordance with relevant guidelines and regulations. Cells were maintained in Dulbecco’s Modified Eagle Medium supplemented with 10% fetal calf serum. After 1 week, the emergent osteoblastic cells were passaged to give passage 1 (P1)^4^.

### Chromatin and DNA extraction

Chromatin was extracted from 1.5 × 10^6^ cells using the ChromaFlash Chromatin Extraction Kit (Epigenetek P2001-100). Protein-free “naked” DNA was isolated from 1.5 × 10^6^ cells treated for 1 hour at 56 °C in lysis buffer (1 M Tris, 0.5 M EDTA, 5 M NaCl, 1% SDS and dH2O, 50 μg/ml proteinase K).

### Telomere chromatin condensation assay (TCCA)

Telomere chromatin compaction was evaluated by determinining the rates of Bal 31 exonuclease, digestion of native and protein-free genomic DNA following a previously described protocol^20^. In brief, 6 μl DNA (500 ng/μl) were mixed with 19 μl of dH_2_O, 25 μl Bal 31 buffer and 1.2 U of Bal 31 nuclease. Digestions were performed at 30 °C for the desired times, followed by inactivation at 65 °C and lysis in 100 μl of lysis buffer containing 50 μg/ml of proteinase K. The length of the protected telomeric repeats was quantified by real-time qPCR using the ΔΔCt method^20^. The dynamics of telomere digestion were fitted to the linear-quadratic curve y^2^ = B0 + B1x + B2x ^2^ (R^2^ ≥ 0.90). A chromatin protection factor (CPF) was calculated from the ratio of the Bal 31 digestion rates of chromatin and naked DNA.

### TERRA expression

Total RNA was extracted with the High Pure RNA Tissue kit (Roche diagnostics). RNA was treated with RNase-Free DNase (Qiagen) for 1 hour, cleaned and concentrated with RNA Clean & Concentrator™ (Zymo Research) with an additional in-column DNase treatment made prior to elution. Specific reverse transcription of TERRA and the U6 control RNA were performed using 10 μM of the TERRA specific oligonucleotide (CCCTAA)_5_ or 1 μM of the U6 oligonucleotide GAACTCGAGTTTGCGTGTCATCCTTGCGC respectively^13^,^21^. A qPCR was performed with 3 μl of this newly synthesized cDNA using GoTaq qPCR Master Mix (Promega), in a final volume of 20 μl. The following primers were used for the amplification of two selected human subtelomeric regions from chromosome 15q (forward: CAGCGAGATTCTCCCAAGCTAAG, reverse: AACCCTAACCACATGAG CAACG) and chromosome 10q (forward: GAATCCTGCGCACCGAGAT, reverse CTGCAC TTGAACCCTGCAATAC). For the quantification of murine TERRA transcripts we used mouse chromosome 18q (forward: AGTTGCAGCATCGGAACG, reverse: CCCTGACCCTAACCCTAACC) and mouse chromosome 10q (forward: GAATCCTGCGCACCGAGAT, reverse CTGCACTTGAACCCTGCAATAC). Data was analyzed with the Lightcycler 480 software release 1.5.0 SP3 software.

### Telomeric RNA-FISH

Telomeric RNA FISH was performed as previously described^13^ with a few modifications. In brief, cells were grown on glass microscope slides and incubated for 7 minutes in ice-cold freshly made CSK buffer (100 mM NaCl, 300 mM sucrose, 3 mM MgCl_2_, 10 mM PIPES pH 7, 0.5% Triton X-100) containing 10 mM Vanadyl Ribonuclease Complex (NEB). Cells were rinsed with PBS, fixed in 4% formaldehyde in 1× PBS (pH 7) for 10 minutes and rinsed in 70% ice-cold ethanol. Cells were dehydrated though an ice-cold ethanol series (70%, 85%, 100% ethanol for 5 minutes each). Slides were air-dried and further dried at 37 °C for 5 minutes. Hybridization with the PNA TelC-Cy3 probe (PANAGEN) was performed with the manufacturer’s hybridization mixture 10 mM NaHPO_4_, 10 mM NaCl, 20 mM Tris with 50% formamide and containing 10 mM Vanadyl Ribonuclease Complex for 18 h at 37 °C. Slides were washed 2 × 5 minutes in 60% formamide in 2× SSc at 37ªC and 1 × 5 min in 2× SSc at 37 °C and 1 × 2 min in 2× SSc at room temperature. DNA was counterstained with 100 ng/ml DAPI in mounting media and covered with a glass cover. RNAse treatment was performed on a separate slide (without Vanadyl Ribonuclease Complex) to probe the RNA nature of the signal. Images were visualized with a Nikon Eclipse E800 epifluorescence microscope and acquired with a CCD camera (KX32ME, Apogee Instruments, Roseville, CA, USA).

### Modulation of Rb1 gene expression

The *RB1* expressing lentivirus (LV-RB1) and knock-down (LV_shRB1) have been previously described^4^,^22^. Stable lentiviral transduction was performed in primary mouse osteoblasts as previously reported^4^. For transient plasmid transfections Ef1-RB1 (overexpression vector) and shRb1 (short hairpin-mediated knock-down vector) were transfected into U2OS cells for 72 hours using FUGENE (Roche) as a carrier. Positive transfections were validated using vector-driven GFP expression and by western blotting of retinoblastoma proteins.

### Functional analysis of the TERRA promoter

The chromosomal region encompassing the TERRA promoter was amplified from human DNA using PCR primers harboring flanking XhoI and HindIII sites (forward:

5′ NNNNNCTCGAGCACCGTGACGTGAGTTTCTG 3′, reverse: 5′ NNNNNAAGCTTGGTGACCCCCAGGTCTGT 3′). The Xho1/HindIII restricted PCR product was ligated into the promoterless pGL3 luciferase expression vector (Promega) created by XhoI and HindIII excision of the original SV40 promoter sequence. This resulted in a TERRA promoter-driven lucifierase reporter vector (vTERRA). A second construct, lacking the 3′ end of the TERRA promoter (vTERRA*) was tested. This truncated version of the TERRA promoter was generated from a chance polymerase misread and is missing the 3′ end of the original construct. This resulted in a truncated version missing the RCEs, located on the minus strand but retaining the three predicted RCEs in the positive strand. The truncated version did not maintain the predicted minimal promoter required for transcription activation.

For the assay of promoter activity we measured luciferase reporter expression and compared this to a transfected control β–Galactosidase reporter. Briefly, 7.5 × 10^5^ U2OS cells were seeded in 6 well plates and transiently transfected 24 hours after plating with 500 ng of plasmid DNA using the X-tremeGENE 9 DNA Transfection Reagent (Roche). To determine the effects of Rb1 on the promoter the *Rb1*-expression modifying plasmid constructs (shRb1, Ef1-RB1), as well as a β-Gal-expressing transfection control vector were transiently transfected along with the reporter construct in U2OS cells. Transfected cells were collected after 72 hours. Promoter activity was quantified using the firefly luciferase glow assay (Pierce Thermo Scientific) and the β-Galactosidase enzymatic assay (Promega), both performed following the manufacturer’s recommendations.

### Chromatin Immunoprecipitation

Assays were performed using the Simple ChIP Enzymatic Chromatin IP Kit (Cell Signaling). Chromatin was fragmented by ultrasound (18 cycles of 20 seconds at 35% amplitude, Bandelin Sonoplus), then incubated with the appropriate primary antibodies overnight at 4 °C. For the subsequent immunoprecipitation the samples were incubated for 2 hours at 4 °C with agarose-bound secondary antibodies, before being washed and separated by centrifugation. Antibody-bound DNA was eluted in H_2_O and subjected to qPCR analysis using the telomeric primers previously described as well as primers flanking the candidate Retinoblastoma Control Element we identified at the 5′ end of the human TERRA promoter region in chromosome 15q homologous to mouse chromosome 18q (Forward: 5′CTGCAACACACGCCCCCC3′ and reverse: 3′TAGCATGTGTCTCTGCGCCT5′). Thermal cycling was performed using 1 cycle at 95 °C for 10 minutes, followed by 50 cycles of 95 °C for 15 seconds, 60 °C for 30 seconds and 72 °C for 45 seconds with a final extension at 72 °C for 2 minutes. ChIP antibodies used for immunoprecipitation were anti-H3K9Ac (Active Motif, Ref. 61251), anti-H4K16Ac (Active Motif, Ref. 39167), anti-H3K4ME3 (Active Motif, Ref. 39159), anti-H3K9ME3 (Active Motif, Ref. 39161), anti-H3K79ME2 (Active Motif, Ref. 39143), anti-H4K20ME3 (Active Motif, Ref. 39671) as well as anti-Rb1 (BD Pharmigen Catalog Number: 554136) and anti-pRb1 S807/811 (Cell Signaling Catalog Number: #8516).

### Identification of the TERRA promoter

The analysis of the transcriptional control sequences of the TERRA repeats was performed using the MatInspector and PromoterInspector and the library of general core promoter elements of the Genomatix Suite software (Genomatix Software GmbH, Munich, Germany)^23^,^24^ and the previously generated Rb1 binding site matrix RCE-Matrix^25^.

## Results

### *Rb1* haploinsufficiency results in altered telomeric chromatin condensation

**Figure 1:**
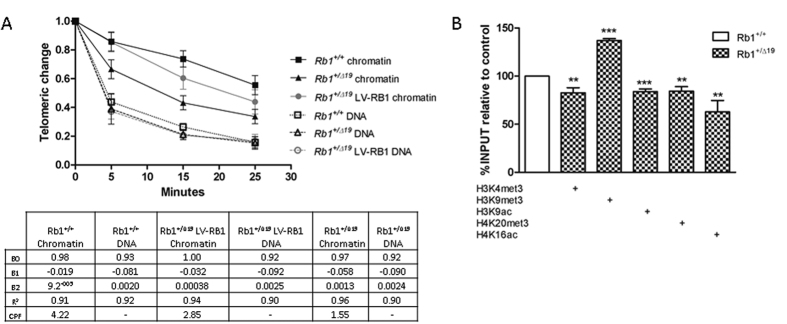
Chromatin modifications in *Rb1*^+/*Δ19*^ osteoblasts. (**A**) Telomere Chromatin Condensation Assay (TCCA) in primary osteoblasts. qPCR based assay to measure the loss of telomeric repeats after enzymatic digestion with the nuclease Bal 31 in *Rb1*^+/+^ chromatin (filled squares), *Rb1*^+/*Δ19*^ chromatin (filled triangles), LV-RB1 chromatin (filled circles in grey), *Rb1*^+/+^ naked DNA (open squares), *Rb1*^+/*Δ19*^ naked DNA (open triangles) and LV-RB1 naked DNA (open circles). Error bars show standard deviation of 2 biological replicates with 3 technical replicates each at passage 6. (**B**) Chromatin immunoprecipitation analysis at the telomere level. The histone modification changes in *Rb1*^+/*Δ19*^ osteoblasts (checked bars) at the telomeres are shown compared to the *Rb1*^+/+^ (open bar). The outcome of the assay is shown for each antibody relative to the input and to the *Rb1*^+/+^ control. Error bars show the mean of 2 biological replicates in 3 consecutive experiments for each antibody. Significances were assessed using Student’s t-test.

The condensation of telomeric chromatin was assayed by Bal 31 nuclese digestion^20^. As expected, no differences were evident in the digestion rates of deproteinated DNA obtained from *Rb1*^+/+^, *Rb1*^+/*Δ19*^ and LV-RB1-recovered *Rb1*^+/*Δ19*^ osteoblastic cells (Fig. 1A). In contrast, the rate of Bal31 digestion of telomeric chromatin was significantly faster in haploinsufficient *Rb1*^+/*Δ19*^cells compared to wild type *Rb1*^+/+^ cells (Fig. 1A). The increased rate of digestion of the telomeres in the absence *Rb1* is consistent with formation of a more open chromatin structure. Over-expression of *RB1* in *LV-RB1-*transduced *Rb1*^+/*Δ19*^*-*haploinsufficent cells reversed the loss of telomeric compaction over at least five passages following transduction (Fig. 1A) filled circles; slope of curve: −0.03222; p = 0.0006).

### Telomeric histone modification is selectively changed in Retinoblastoma haploinsufficiency

Whilst the level of H3K4me3 modification of telomeric histone was unaffected by retinoblastoma status (p = 0.5016), the representation of both H3K9me3 and H3K9ac in the telomeric chromatin was significantly increased in haploinsufficient (*Rb1*^+/*Δ19*^) cells (Fig. 1B). The increased abundance of these histones was accompanied by significantly decreased levels of H3K79me2, H4K20me3 and H4K16ac in the *Rb1*^+/*Δ19*^osteoblasts.

### Rb1 modulates expression of the telomeric long non-coding RNA TERRA

**Figure 2:**
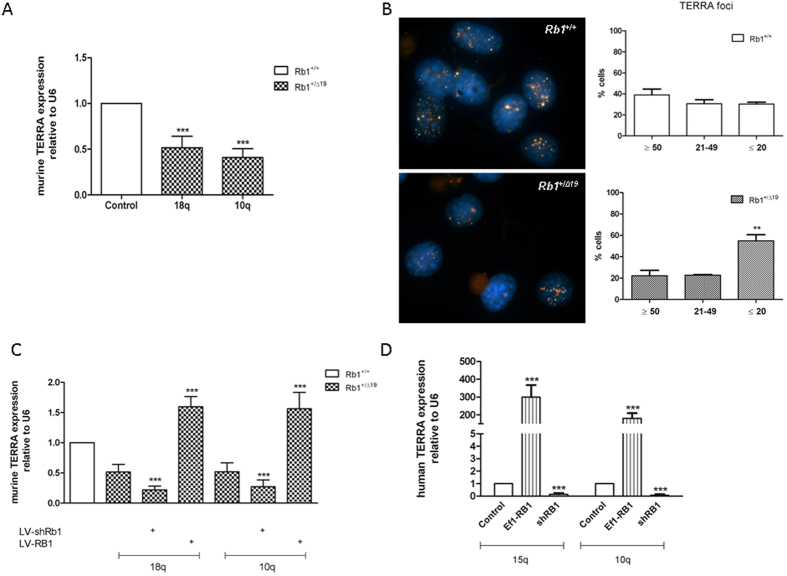
TERRA steady levels study and endogenous modulation dependent upon Rb1 cellular levels. (**A**) Significant reduction of the mouse TERRA transcriptional levels on chromosomes 18q and 10q were found in *Rb1*^+/*Δ19*^ osteoblasts (squared) when compared to their wild-type counterparts (filled column). Error bars show the SD of 2 biological replicates with 2 technical replicates each. RNA-FISH of TERRA in murine osteoblast cells reveals different strengths of labeling of TERRA foci between wild-type and *Rb1* haploinsufficient cells. The number of foci were counted in 250 cells of each genotype. Upper panels show a representative image of the cell nuclei with TERRA foci present in wild-type osteoblasts (left) and distribution histogram (right). Lower panels show a representative image of the TERRA foci present in *Rb1* haploinsufficient osteoblasts (lower left) and the distribution histogram (lower right). Mean (bars) and SDs (error bars) were obtained from two technical replicates. Yellow is the TERRA signal stained with a strand specific telomeric probe labeled with Cy3 and blue is the DAPI staining the cell nucleus. (**C**) Endogenous TERRA expression appears modulated by Rb1 levels on murine chromosomes 18q and 10q. A reduction of the Rb1 protein with lentiviral infection expressing *shRB1* led to a further decrease of TERRA levels in the *Rb1*^+/*Δ19*^ osteoblasts. A lentiviral construct (LV-RB1) overexpressing *Rb1* rescued the haploinsufficient phenotype leading to an increase in endogenous TERRA expression. Error bars show the SD of 2 biological replicates with 2 technical replicates each (**D**) Endogenous TERRA expression in human U2OS cells was modulated by Rb1 expression levels on chromosomes 15q and 10q. TERRA levels were reduced after transient transfection with a shRB1 vector. In the same manner transient overexpression of LV-RB1 led to a significant increase of TERRA levels in the human osteosarcoma cell line. Error bars show the SD of 3 technical replicates. Significance of all experiments assessed with one-way ANOVA.

Steady state levels of lncRNA TERRA transcripts from the subtelomeric regions of both murine chromosomes 18q and chromosome 10q were reduced by almost half in *Rb1* haploinsufficient cells (Fig. 2A). TERRA RNA-FISH revealed a significant decrease of TERRA foci in *Rb1*^+/*Δ19*^ mouse osteoblasts when compared to their wild-type counterparts (Fig. 2B). For further confirmation, Rb1 levels were modulated *in vitro* to assess Rb1 influence on endogenous TERRA transcription. A further reduction of the residual Rb1 expression in haploinsufficient cells, using lentivirally-driven shRNA expression (LV-shRB1), further reduced the endogenous TERRA levels. Conversely a lentiviral-mediated increase in Rb1 levels in the haploinsufficient cells resulted in an increase of the steady state levels of endogenous TERRA (Fig. 2C). To assess whether this modulation of TERRA expression also occurs in human cells, RB1 proficient human U2OS cells were transiently transfected with the shRB1 knock-down vector (Supplementary 1) and a decrease in the levels of the endogenous TERRA originating from both of the investigated human chromosomes (Chr15q, and Chr10q) was observed (Fig. 2D). The same effect was also seen on chromosomes XpYp (Supplementary 2). Furthermore, the overexpression of RB1 driven by the Ef1-RB1 vector (Supplementary 1) also elicited a significant increase in TERRA transcripts in the U20S cells in the three chromosomes assayed (Fig. 2D and Supplementary 2).

### The predicted human TERRA promoter sequence confers Rb1-dependent expression onto a luciferase reporter *in vitro*

*In silico* analysis of the subtelomeric region of human chromosome 15q identified a putative promoter proximal to the transcriptional start site of TERRA gene.

**Figure 3:**
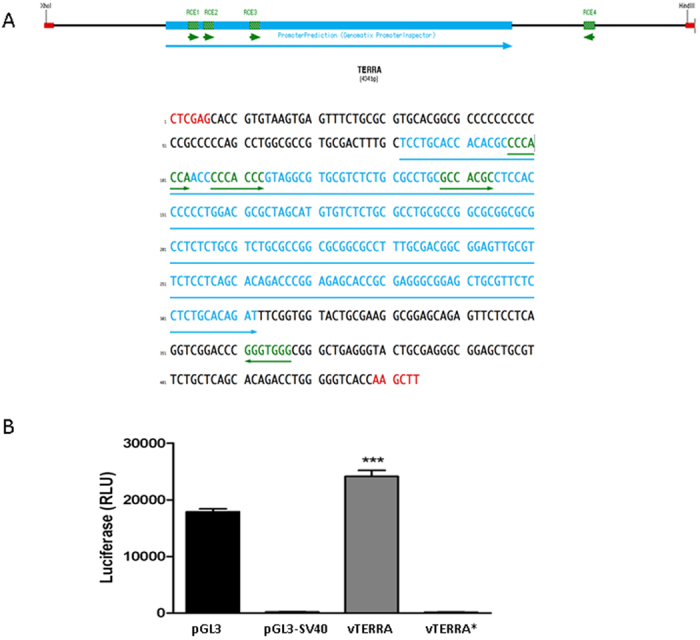
*In silico* promoter analysis of the 434 bp TERRA sequence from the subtelomere of human chromosome 15q. (**A**) The PromoterInspector (Genomatix) predicted core promoter is displayed in blue and underlined with a blue bar. Matches to the MatInspector (Genomatix) predicted Retinoblastoma control elements (RCEs) are marked in green. The strand orientations are indicated by the underlining green arrows. The 5′ Xho II and the 3′ HindIII restriction sites recognition sequences are shown in red. (**B**) Luciferase expression of all the constructs cloned into pGL3 used in this study. The pGL3 vector was used as a positive control (black bar). The promotorless vector (pGL3-SV40) was unable to drive luciferase expression and used as negative control. The full length TERRA promoter vector (vTERRA) drove luciferase expression (grey bar). The expression under this promoter was significantly stronger than the SV40 promoter present in the pGL3 control vector. The truncated version of the TERRA promoter (vTERRA*) was not able to drive luciferase expression. RLU stands for relative luciferase units (relative to β-Galactosidase). Error bars show the SD of 3 technical replicates.

This sequence includes a CpG island predicted by EMBOSS Cpgplot, and the Promoter Inspector and is located 434 bp upstream of the start of repetitive telomeric sequences, harbours a minimal promoter region of 232 bp between base pairs 81 and 312 (Fig. 3A) MatInspector identified 4 matches with an RCE (Retinoblastoma Control Elements)-Matrix generated using previously published RCE sequences^26^. Three of the identified RCEs were located on the plus strand (97-103 cCCACca; 107-113 cCCACca; 138-144 gCCACgc), one on the minus strand (367-361 cCCACcc). All of the predicted RCEs located on the + strand fell within the minimal promoter region (marked in blue).

Two TERRA inserts were cloned into a luciferase reporter vector replacing its own promoter sv40. The full-length minimal TERRA promoter unit (434 bp) named vTERRA and a chance PCR polymerase misread truncated version (317 bp) named vTERRA* (Supplementary 3). When inserted into a luciferase reporter vector vTERRA, harbouring the predicted TERRA promoter was sufficient to drive luciferase expression upon transient transfection into U2OS cells (Fig. 3B). The truncated TERRA promoter vTERRA* was unable to drive luciferase expression (Fig. 3B).

**Figure 4:**
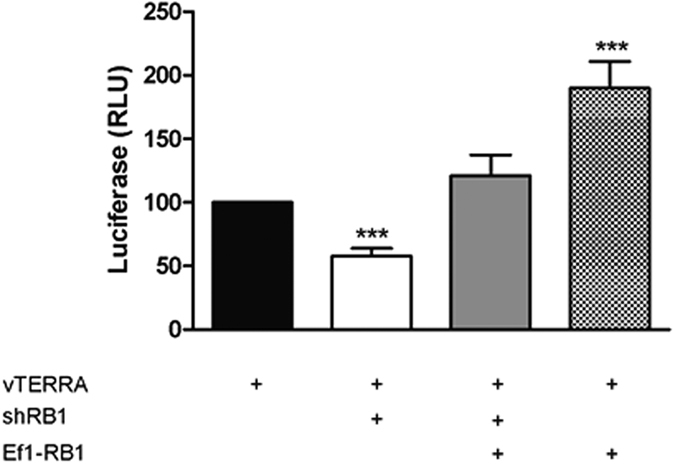
*In vitro* TERRA promoter driven luciferase expression is modulated by Rb1 levels. U2OS transient co-transfections of the TERRA promoter vector (vTERRA) with the RB1 knocking down vector (shRb1) or the overexpressing RB1 vector (Ef1-RB1) led to *in vitro* modulated luciferase expression depending on the Rb1 levels. Error bars show the standard deviation of 3 technical replicates harvested 72 hours after transfection. RLU stands for relative luciferase units (relative to β-Galactosidase). Error bars show the SD of 3 technical replicates. Significances assessed using the Student’s t-test. RLU stand for relative light units calculated relative to β-Galactosidase.

Reduction of *RB1* expression in U2OS cell using the *shRB1* vector reduced transcription from the vTERRA luciferase reporter; conversely *RB1* overexpression resulted in an increased reporter gene transcription (Fig. 4). These RB1 modulations had no impact on the activity of a control pGl3 promoter (Supplementary 4).

### Rb1 modulates the transcriptional activity of the TERRA promoter by physical interaction

**Figure 5:**
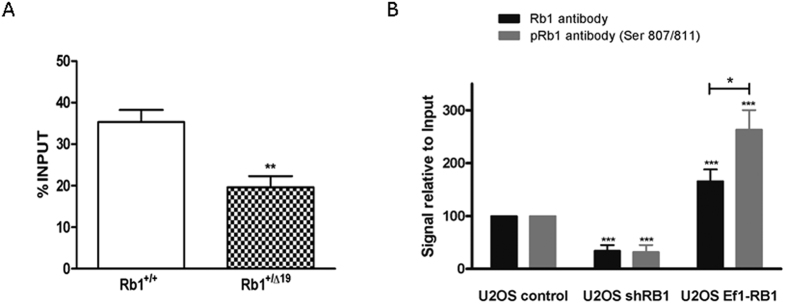
Chromatin immunoprecipitation evidence the spatial relationship between Rb1 and the TERRA promoter in mouse and human cells. (**A**) ChIP with anti-Rb1 in *Rb1*^+/+^ and *Rb1*^+/*Δ19*^ murine osteoblasts showed that *Rb1* haploinsufficiency led to a significant reduction of the endogenous TERRA promoter sequence recovered from murine cells. Error bars show the SD of 3 biological replicates. Student’s t-test was used to assess the significance. (**B**) Transient transfections in U2OS show that RB1 levels influence the amount of endogenous TERRA promoter recovered after immunoprecipitation with anti-RB1 and anti-pRb1 (Ser 807/811). Error bars show the mean of 3 technical replicates for each antibody. Significance was assessed with the ANOVA test and Student t-test for comparison among the two different transfected cells.

Chromatin immunoprecipitations of genomic DNA using an anti-Rb1 antibody were performed in order to quantify the amount of endogenous TERRA promoter sequence recovered bound to the Rb1 protein. Enrichment for TERRA promoter sequences (Fig. 5A) was seen on the sequence identified on murine chromosome 18q after pull down with Rb1 antibody. Interestingly, the amount of promoter DNA recovered from haploinsufficient mouse *Rb1*^+/*Δ19*^cells was almost half of that recovered from the wild-type counterparts. Transient overexpression of RB1 in U2OS cells further increased the amount of immunoprecipitable TERRA promoter sequences (Fig. 5B) on the sequence identified on chromosome 15q, whilst a reduction of RB1 by shRNA knock-down in U2OS cells reduced the recovery of the promoter sequence (Fig. 5B). Importantly, in U2OS cells that were transiently overexpressing RB1 the TERRA promoter could be efficiently immunoprecipitated using an antibody directed against phosphorylated Rb1 (Ser 807/811) that fails to detect non-phosphorylated RB1.

## Discussion

Effective telomere capping of chromosomal ends requires an adequate length of telomere repeats and a highly condensed heterochromatic state^9^,^27^,^28^. The loss of either feature impairs protection of the ends and increases the risk of carcinogenesis, presumably through erroneous recognition of chromosome ends as double strand breaks leading to inappropriate DNA double strand break repair^29^,^30^.

This would result in the illicit end-to-end joining of chromosomes and cycles of breakage and fusion. Pathological telomere shortening and decondensation may result from alterations in any of the components tasked with maintain telomere homeostasis, i.e. defects affecting the sheltering complex, the long non-coding RNA TERRA, or the epigenetic marks responsible for initiating and maintaining higher ordered chromatin organization at the telomeres^10^,^17^,^31^,^32^.

A haploinsufficient mutation of the tumour suppressor gene *Rb1* has been shown to be sufficient to trigger telomere attrition, leading to genomic instability in primary mouse osteoblasts^4^. On such study a faster attrition of the telomere ends and an increase in acentric fragments, telomere fragments and anaphase bridges were discovered in Rb1^+/Δ19^ when compared to their wild-type counterparts.

In mouse embryonic fibroblasts (MEFs) the removal of all three Retinoblastoma family members (a triple knockout of RB1, RBL1 and RBL2) also increased genomic instability, although paradoxically this was reported to be associated with telomeric elongation^33^ despite the loss of H4K20me3 at telomeric chromatin that would indicate relaxation of normally condensed telomeric DNA^34^.

The increase in telomere sensitivity to nuclease digestion that we now report in Rb1-haploinsufficien osteoblast cells occurs rapidly during passages, and can be equally rapidly restored by recovery of Rb1 expression. This could point to a system where improper telomeric chromatin architecture, drives the loss of telomere length. The telomeric decondensation observed in Rb1 deficiency is accompanied by a quantitative shift in the pattern of histone modifications at the telomeres (increased H3K9me3 and H3K9ac and reduced H3K79me2, H4K20me3 and H4K16ac) that is consistent with the observed relaxation of the chromatin. Interestingly, this pattern of histone alteration has been previously recognized as a landmark of malignant transformation. We now suggest that it is also an indicator of telomere-dependent genomic instability, which may well precede acquisition of malignancy^35^. Previous reports suggest that the observed changes in H3K9me3 and H3K9ac are mutually exclusive^36^. We can only speculate that there are spatially segregated patterns of histone modifications along the telomeres.

Whilst the observed decrease in H4K20me3 and increase in Histone-3 acetylation are consistent with a reduced condensation of telomeric chromatin they do not explain the elongation of telomeres reported in MEFs deficient in all three Rb family members^34^. This could be explained if the histone architecture initiates a shift in the balance between TERRA dependent telomerase activity and the alternative lengthening of telomeres (ALT) pathway. The absence of all Rb family proteins may trigger lengthening of telomeres by either the ALT pathway^14^,^37^,^38^ or by reactivating telomerase^39^.

Overall, our results support the hypothesis that Rb1 regulates the condensed state of telomeres both genetically and epigenetically. This is brought about via Rb1-dependent transcription of the TERRA long non-coding RNA, thereby ensuring appropriate telomeric chromatin architecture. Activation of TERRA transcription has also been reported to be activated by local depletion of TRF2 40. It is not impossible that TRF2 deficiency increases Rb1 access to the TERRA promoter via local shelterin-dependent chromatin relaxation. Down-regulation of mouse Chr18q-TERRA reportedly results in an increase in telomere damage^10^, which is consistent with the increased genomic instability we observed in Rb1 haploinsufficient cells^4^.

The 434 bp region studied on human chromosome 15q is located immediately adjacent to the telomeric tract and contains the 232 bp in silico predicted.minimal promoter. This chromosomal location is similar to that of other subtelomere promoter areas previously reported, such as for chromosomes XpYp where the TSSs was found 250 bp upstream the telomeric tract^8^.

Nergadze *et al*.^41^ reported that several subtelomeres share a conserved repetitive region that comprises, in a centromere to telomere direction, a 61 bp repeat element, a 29 bp repeat element and a 37 bp repeat element, altogether referred to as 61-29-37 repeats. The 29 bp and the 37 bp repeats form a CpG island which they found essential for TERRA transcription^41^. Therefore, the promoter unit reported in our study could be part of a broader regulatory region.

We demonstrate that TERRA transcription is under the control of Rb1 but there is a formal possibility that this effect is restricted to the two chromosomes (Chr 10q, Chr 18q) studied. However the available evidence suggests a global TERRA reduction as the RNA-FISH experiments performed in this study showed an Rb1 expression-dependent reduction in TERRA foci. Nevertheless, as TERRA transcripts from other chromosomes become available in mice it will be interesting to determine if there is differential regulation.

The co-precipitation of phospho-Rb1 and the TERRA promoter suggests that the cyclin-dependent kinase mediated phosphorylation of Rb1 may serve to cycle phospho-Rb1 from E2F complexes to the TERRA promoter. This would allow temporal coordination of the activation of E2F-dependent genes required for G1/S transit with the opening of the telomere chromatin for replication by TERRA. Intriguingly, human herpesviruses HSV1 have evolved the capacity to target both Rb1-E2F^42^ and TERRA transcription^43^ as part of their life cycle.

As shown for other long non-coding RNA molecules, TERRA is able to form local DNA:RNA hybrids^7^,^18^. At the telomeres this may serve as a scaffold for protein-protein and protein-DNA interactions. It is therefore possible that TERRA acts to initiate or sustain the shelterin complexes essential for proper functional chromatin formation^9^. Indeed, TERRA has been shown to facilitate binding of TRF1 and TRF2^44^, the recruitment of the heterochromatic proteins origin recognition protein (ORC), heterochromatin protein 1 (HP1)^44^, the histone methyltransferase SUV39H1 and MORF4L2, a component of the Nua2 histone acetylase complex^44^. The observation that Rb1 may itself shuttle histone-modulating SUV proteins to the telomeres^33^,^36^ is consistent with our contention that loss of Rb1 alters histone modification.

We suggest that the role of *Rb1* as a tumour suppressor in osteosarcoma will be exerted by both canonical Rb1 activity on cell cycle transit, and telomere homeostasis. This may be the mechanism by which germ line mutations of *Rb1* so effectively predispose to cancer^45^. Other sarcoma-type tumours may also inactivate TERRA, albeit through a different biological mechanism. Thus, the RGG-containing proteins overexpressed in Ewing’s sarcoma (EWS) and liposarcoma (TLS) are able to interact with the G-quadruplex sequences of TERRA^46^,^47^. It will be informative to determine if other RGG-containing proteins, such as MRE11 and P53 involved in the DNA damage response and the miRNA processing exonuclease DROSHA, are also able to bind TERRA. A second sequalae of retinoblastoma deficiency involving TERRA may be infertility, with reduced spermatogenesis being associated with both Rb1 loss^48^ and decreased TERRA transcription^36^. Thus, telomere dysfunction through abnormal epigenetic modifications could be a more general mechanism of cellular pathology.

## Additional Information

**How to cite this article**: Gonzalez-Vasconcellos, I. *et al*. The *Rb1* tumour suppressor gene modifies telomeric chromatin architecture by regulating TERRA expression. *Sci. Rep.* **7**, 42056; doi: 10.1038/srep42056 (2017).

**Publisher's note:** Springer Nature remains neutral with regard to jurisdictional claims in published maps and institutional affiliations.

## Supplementary Material

Supplementary Material

## References

[b1] Eng C. . Mortality from second tumors among long-term survivors of retinoblastoma. J. Natl. Cancer Inst. 85, 1121–8 (1993). 8320741 10.1093/jnci/85.14.1121

[b2] Kansara M., Teng M. W., Smyth M. J. & Thomas D. M. Translational biology of osteosarcoma. Nat. Rev. Cancer 14, 722–35 (2014). 25319867 10.1038/nrc3838

[b3] Evans R. D. The radium standard for boneseekers–evaluation of the data on radium patients and dial painters. Health Phys. 13, 267–78 (1967). 5231341 10.1097/00004032-196703000-00004

[b4] Gonzalez-Vasconcellos I. . Rb1 haploinsufficiency promotes telomere attrition and radiation-induced genomic instability. Cancer Res. 73, 4247–55 (2013). 23687339 10.1158/0008-5472.CAN-12-3117

[b5] Gonzalez-Vasconcellos I. . Differential effects of genes of the Rb1 signalling pathway on osteosarcoma incidence and latency in alpha-particle irradiated mice. Radiat. Environ. Biophys. 50, 135–141 (2011). 21063720 10.1007/s00411-010-0339-4

[b6] Bailey S. M. & Murnane J. P. Telomeres, chromosome instability and cancer. Nucleic Acids Res. 34, 2408–2417 (2006). 16682448 10.1093/nar/gkl303 PMC1458522

[b7] Cusanelli E. & Chartrand P. Telomeric noncoding RNA: telomeric repeat-containing RNA in telomere biology. Wiley Interdiscip. Rev. RNA 5, 407–19 (2014). 24523222 10.1002/wrna.1220

[b8] Farnung B. O., Giulotto E. & Azzalin C. M. Promoting transcription of chromosome ends. Transcription 1, 140–143 (2010). 21326888 10.4161/trns.1.3.13191 PMC3023574

[b9] Cusanelli E. & Chartrand P. Telomeric repeat-containing RNA TERRA: a noncoding RNA connecting telomere biology to genome integrity. Front. Genet. 6, 143 (2015). 25926849 10.3389/fgene.2015.00143 PMC4396414

[b10] López de Silanes I. . Identification of TERRA locus unveils a telomere protection role through association to nearly all chromosomes. Nat. Commun. 5, 4723 (2014). 25182072 10.1038/ncomms5723 PMC4164772

[b11] Atianand M. K. . A Long Noncoding RNA lincRNA-EPS Acts as a Transcriptional Brake to Restrain Inflammation. Cell 165, 1672–85 (2016). 27315481 10.1016/j.cell.2016.05.075 PMC5289747

[b12] Postepska-Igielska A. . LncRNA Khps1 Regulates Expression of the Proto-oncogene SPHK1 via Triplex-Mediated Changes in Chromatin Structure. Mol Cell. 60, 626–36 (2015). 26590717 10.1016/j.molcel.2015.10.001

[b13] Azzalin C. M., Reichenbach P., Khoriauli L., Giulotto E. & Lingner J. Telomeric repeat containing RNA and RNA surveillance factors at mammalian chromosome ends. Science 318, 798–801 (2007). 17916692 10.1126/science.1147182

[b14] Arora R. . RNaseH1 regulates TERRA-telomeric DNA hybrids and telomere maintenance in ALT tumour cells. Nat. Commun. 5, 5220 (2014). 25330849 10.1038/ncomms6220 PMC4218956

[b15] Balk B. . Telomeric RNA-DNA hybrids affect telomere-length dynamics and senescence. Nat. Struct. Mol. Biol. 20, 1199–205 (2013). 24013207 10.1038/nsmb.2662

[b16] Zhang W. . A Werner syndrome stem cell model unveils heterochromatin alterations as a driver of human aging. Science 348, 1160–3 (2015). 25931448 10.1126/science.aaa1356 PMC4494668

[b17] Schoeftner S. & Blasco M. A. A ‘higher order’ of telomere regulation: telomere heterochromatin and telomeric RNAs. EMBO J. 28, 2323–2336 (2009). 19629032 10.1038/emboj.2009.197 PMC2722253

[b18] O’Leary V. B. . PARTICLE, a Triplex-Forming Long ncRNA, Regulates Locus-Specific Methylation in Response to Low-Dose Irradiation. Cell Rep. 11, 474–85 (2015). 25900080 10.1016/j.celrep.2015.03.043

[b19] Siggelkow H. . Development of the osteoblast phenotype in primary human osteoblasts in culture: comparison with rat calvarial cells in osteoblast differentiation. J. Cell. Biochem. 75, 22–35 (1999). 10462701

[b20] Gonzalez-Vasconcellos I., Alonso-Rodríguez S., López-Baltar I. & Fernández J. L. Telomere Chromatin Condensation Assay (TCCA): A novel approach to study structural telomere integrity. Mutat. Res. 771, 51–5 (2015). 25771980 10.1016/j.mrfmmm.2014.12.004

[b21] Smirnova A. . TERRA Expression Levels Do Not Correlate with Telomere Length and Radiation Sensitivity in Human Cancer Cell Lines. Front. Oncol. 3, 115 (2013). 23717814 10.3389/fonc.2013.00115 PMC3650684

[b22] Höfig I. . Poloxamer synperonic F108 improves cellular transduction with lentiviral vectors. J. Gene Med. 14, 549–60 (2012). 22887595 10.1002/jgm.2653

[b23] Quandt K., Frech K., Karas H., Wingender E. & Werner T. MatInd and MatInspector: new fast and versatile tools for detection of consensus matches in nucleotide sequence data. Nucleic Acids Res. 23, 4878–84 (1995). 8532532 10.1093/nar/23.23.4878 PMC307478

[b24] Werner T. Computer-assisted analysis of transcription control regions. Matinspector and other programs. Methods Mol. Biol. 132, 337–49 (2000). 10547845 10.1385/1-59259-192-2:337

[b25] Rosemann M. . A Rb1 promoter variant with reduced activity contributes to osteosarcoma susceptibility in irradiated mice. Mol. Cancer 13, 182 (2014). 25092376 10.1186/1476-4598-13-182 PMC4237942

[b26] Banchio C., Lingrell S. & Vance D. E. Sp-1 binds promoter elements that are regulated by retinoblastoma and regulate CTP:phosphocholine cytidylyltransferase-alpha transcription. J. Biol. Chem. 282, 14827–35 (2007). 17384411 10.1074/jbc.M700527200

[b27] Azzalin C. M. & Lingner J. Telomere functions grounding on TERRA firma. Trends Cell Biol. 25, 29–36 (2015). 25257515 10.1016/j.tcb.2014.08.007

[b28] Michishita E. . SIRT6 is a histone H3 lysine 9 deacetylase that modulates telomeric chromatin. Nature 452, 492–6 (2008). 18337721 10.1038/nature06736 PMC2646112

[b29] Tang J., Georgescu W., Deschamps T., Yannone S. M. & Costes S. V. In 75–93 (Springer International Publishing, doi: 10.1007/978-3-319-12136-9_4 (2015).

[b30] Khanna K. K. & Jackson S. P. DNA double-strand breaks: signaling, repair and the cancer connection. Nat. Genet. 27, 247–54 (2001). 11242102 10.1038/85798

[b31] Vajen B., Thomay K. & Schlegelberger B. Induction of Chromosomal Instability via Telomere Dysfunction and Epigenetic Alterations in Myeloid Neoplasia. Cancers (Basel). 5, 857–74 (2013). 24202323 10.3390/cancers5030857 PMC3795368

[b32] Stimpson K. M., Sullivan L. L., Kuo M. E. & Sullivan B. A. Nucleolar organization, ribosomal DNA array stability, and acrocentric chromosome integrity are linked to telomere function. PLoS One 9, e92432 (2014). 24662969 10.1371/journal.pone.0092432 PMC3963894

[b33] Schoeftner S. & Blasco M. A. A ‘higher order’ of telomere regulation: telomere heterochromatin and telomeric RNAs. EMBO J. 28, 2323–36 (2009). 19629032 10.1038/emboj.2009.197 PMC2722253

[b34] Gonzalo S. . Role of the RB1 family in stabilizing histone methylation at constitutive heterochromatin. Nat. Cell Biol. 7, 420–8 (2005). 15750587 10.1038/ncb1235

[b35] Fraga M. F. . Loss of acetylation at Lys16 and trimethylation at Lys20 of histone H4 is a common hallmark of human cancer. Nat. Genet. 37, 391–400 (2005). 15765097 10.1038/ng1531

[b36] Reig-Viader R. . Telomere homeostasis is compromised in spermatocytes from patients with idiopathic infertility. Fertil. Steril. 102, 728–738 e1 (2014). 24996497 10.1016/j.fertnstert.2014.06.005

[b37] Henson J. D. . DNA C-circles are specific and quantifiable markers of alternative-lengthening-of-telomeres activity. Nat. Biotechnol. 27, 1181–5 (2009). 19935656 10.1038/nbt.1587

[b38] Henson J. D. . A robust assay for alternative lengthening of telomeres in tumors shows the significance of alternative lengthening of telomeres in sarcomas and astrocytomas. Clin. Cancer Res. 11, 217–25 (2005). 15671549

[b39] Redon S., Reichenbach P. & Lingner J. The non-coding RNA TERRA is a natural ligand and direct inhibitor of human telomerase. Nucleic Acids Res. 38, 5797–806 (2010). 20460456 10.1093/nar/gkq296 PMC2943627

[b40] Okamoto K. . A two-step mechanism for TRF2-mediated chromosome-end protection. Nature 494, 502–5 (2013). 23389450 10.1038/nature11873 PMC3733551

[b41] Nergadze S. G. . CpG-island promoters drive transcription of human telomeres. RNA 15, 2186–2194 (2009). 19850908 10.1261/rna.1748309 PMC2779677

[b42] Herpes simplex virus type 1 infection imposes a G(1)/S block in asynchronously growing cells and prevents G(1) entry in quiescent cells. Virology 267, 335–49 (2000). 10662629 10.1006/viro.1999.0147

[b43] Deng Z. . HSV-1 remodels host telomeres to facilitate viral replication. Cell Rep. 9, 2263–78 (2014). 25497088 10.1016/j.celrep.2014.11.019 PMC4356630

[b44] Deng Z., Norseen J., Wiedmer A., Riethman H. & Lieberman P. M. TERRA RNA binding to TRF2 facilitates heterochromatin formation and ORC recruitment at telomeres. Mol. Cell 35, 403–13 (2009). 19716786 10.1016/j.molcel.2009.06.025 PMC2749977

[b45] Dommering C. J. . RB1 mutations and second primary malignancies after hereditary retinoblastoma. Fam. Cancer 11, 225–33 (2012). 22205104 10.1007/s10689-011-9505-3 PMC3365233

[b46] Takahama K. & Oyoshi T. Specific binding of modified RGG domain in TLS/FUS to G-quadruplex RNA: tyrosines in RGG domain recognize 2′-OH of the riboses of loops in G-quadruplex. J. Am. Chem. Soc. 135, 18016–9 (2013). 24251952 10.1021/ja4086929

[b47] Takahama K. . Regulation of Telomere Length by G-Quadruplex Telomere DNA- and TERRA-Binding Protein TLS/FUS. Chem. Biol. 20, 341–350 (2013). 23521792 10.1016/j.chembiol.2013.02.013

[b48] Yang Q.-E., Gwost I., Oatley M. J. & Oatley J. M. Retinoblastoma protein (RB1) controls fate determination in stem cells and progenitors of the mouse male germline. Biol. Reprod. 89, 113 (2013). 24089198 10.1095/biolreprod.113.113159 PMC4434987

