# Supplementary material for: The Rb1 tumour suppressor gene modifies telomeric chromatin architecture by regulating TERRA expression
Source: Sci Rep. 2017 Feb 7;7:42056. doi: 10.1038/srep42056 (PMC5294645; doi:10.1038/srep42056)
Supplement: Supplementary Material [file srep42056-s1.pdf]

The *Rb1* tumour suppressor gene modifies telomeric chromatin architecture by regulating TERRA expression.

I. Gonzalez-Vasconcellos[a], R. Schneider[b], Natasa Anastasov[C], S. Alonso-Rodriguez[a], B. Sanli-Bonazzi[c], JL Fernández[a,d], MJ Atkinson[c*]

Supplementary 1: **A**. GFP signal in the U2OS cells transfected with the Rb1 knock down vector (shRB1) or the overexpressing vector (Ef1-RB1), 72h post transfection. **B**. Western blot and its quantification performed with the Rb1 antibody form BD to assess the amount of Rb1 protein after transient transfections.

Supplementary 2: Q-PCR quantification showed the endogenous TERRA expression in human U2OS cells modulated by Rb1 expression levels on chromosome XpYp. Control bar is the data for the untransfected U2OS cells. Ef1-RB1 bar shows the data for the U2OS transfected with the RB1 over-expressing vector and the shRB1 bar shows the data for the U2OS transfected with the RB1 knock-down vector. Error bars show the SD of 3 technical replicates. Significance of the experiment was assessed with one-way ANOVA.

Supplementary 3: Cloning strategy of the TERRA promoter into a luciferase reporter vector (pGl3) and constructs formed. Cloning was performed using the XhoI site (*) and Hind III site (**) to remove the sv40 promoter and replace it with the TERRA promoter predicted sequence (vTERRA). The vTERRA* truncated construct lacking the 3´end was randomly obtained during the cloning process.

Supplementary 4: *In vitro* luciferase expression driven by the sv40 promoter (pGl3) is not modulated by RB1 levels in transient co-transfections with either shRB1 or Ef1-RB1. Error bars show the standard deviation of three technical replicates 72 hours after transfection. RLU stands for relative luciferase units (relative to β-Galactosidase).

Supplementary 5: All primers used in this study.

Supplementary 1

A

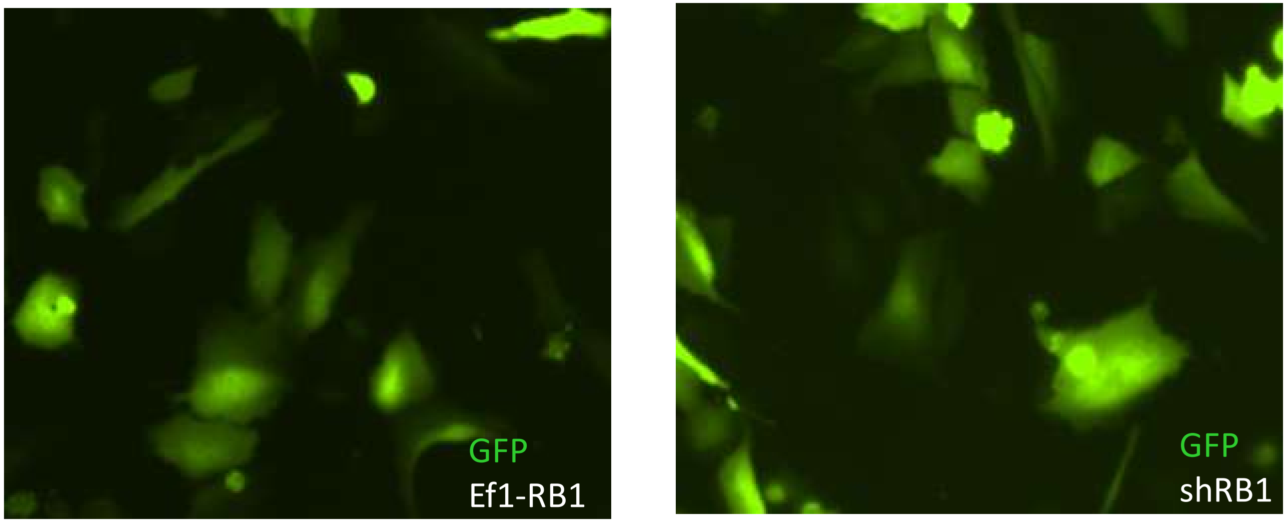

B

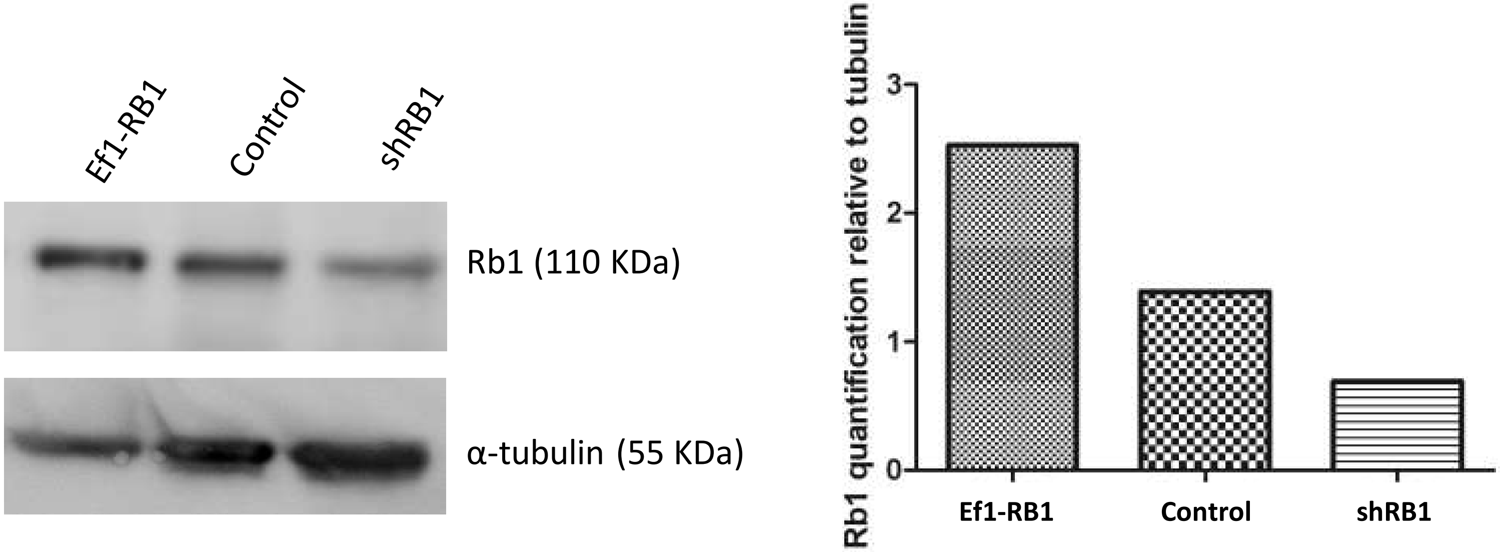

Supplementary 2

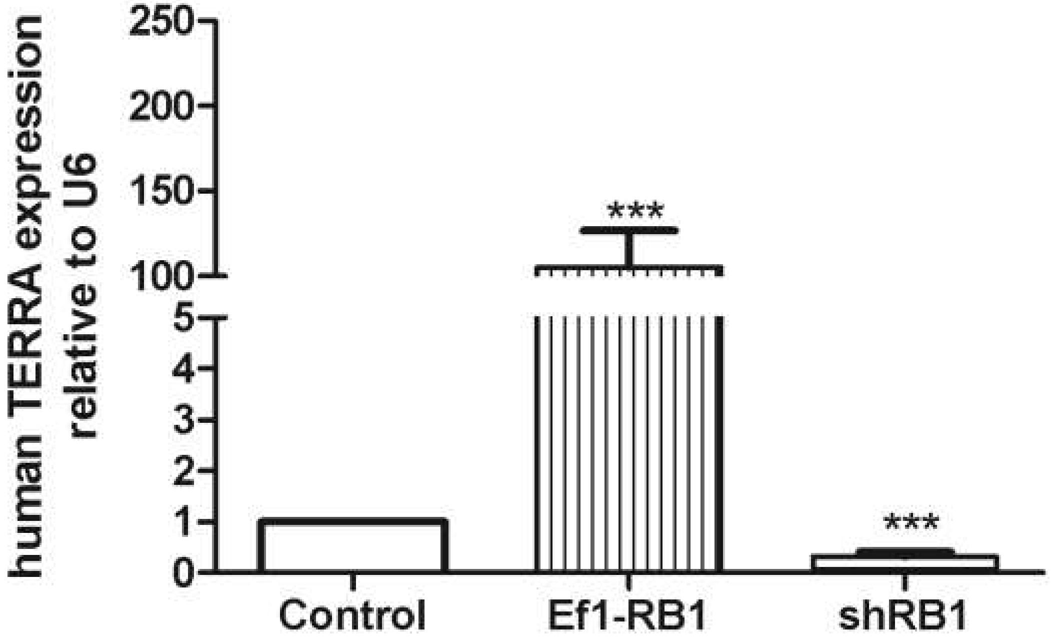

Supplementary 3

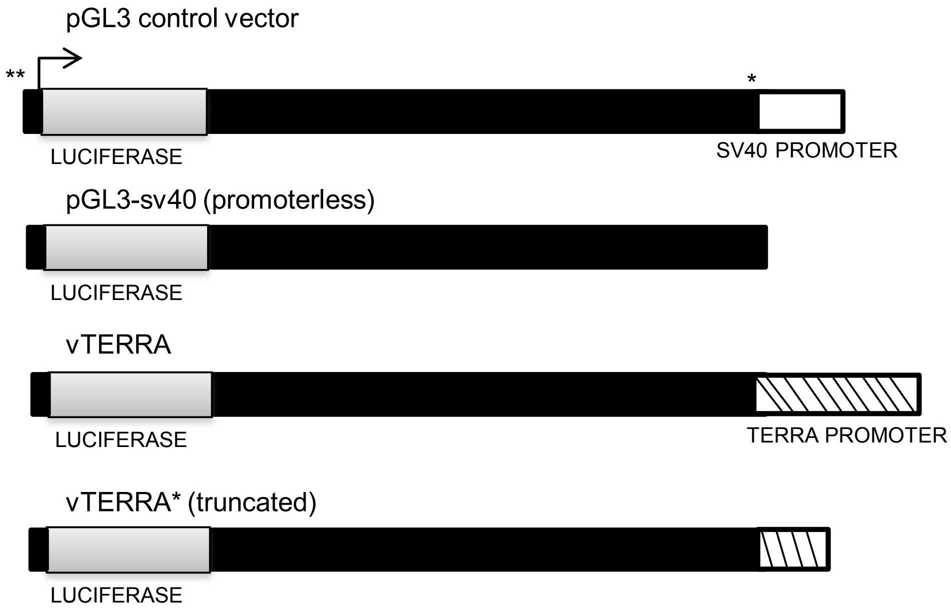

Supplementary 4

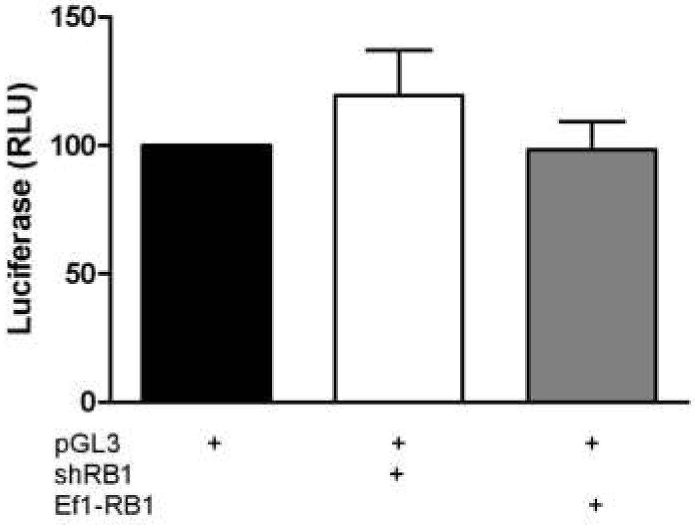

Supplementary 5

Telo fwd	CGGTTTGTTTGGGTTTGGGTTTGGGTTTGGGTTTGGGT
Telo rev	GGCTTGCCTTACCCTTACCCTTACCCTTACCCTTACCCT
36B fwd	CAGCCAGTGGGAAGGTGTAATCC
36B rev	CCCATTCTATCATCAACGGGTACAA
TERRA_ChIP fw	CTGCAACACACGCCCCCC
TERRA_ChIP rv	TAGCATGTGTCTCTGCGCCT
XhoI TERRA fw	NNNNNCTCGAGCACCGTGACGTGAGTTTCTG
HindIII TERRA rev	NNNNNAAGCTTGGTGACCCCCAGGTCTGT
Ch18Mm fw	AGTTGCAGCATCGGAACG
Ch18Mm rv	CCCTGACCCTAACCCTAACC
Dmnt1m fw	GCCTCGGTTCTTCCTTCTGG
Dmnt1m rev	CAGCCTGGAGCACACCAAAG
Dmnt3a fw	CAGCGTCACACAGAAGCATATCC
Dmnt3a rev	GGTCCTCACTTTGCTGAACTTG
ChXpYp fwd	GCAAAGAGTGAAAGAACGAAGCTT
ChXpYp rev	CCCTCTGAAAGTGGACCAATCA
Ch10q fw	GAATCCTGCGCACCGAGAT
Ch10q rev	CTGCACTTGAACCCTGCAATAC
U6 forward	GGAATCTAGAACATATACTAAAATTGGAAC
U6 reverse	GAACTCGAGTTTGCGTGTCATCCTTGCGC
Chr 15 Hm fw	CAGCGAGATTCTCCCAAGCTAAG
Chr 15 Hm rv	AACCCTAACCACATGAGCAACG
